# A study of suicidal ideation in acute ischemic stroke patients

**DOI:** 10.1186/s12955-014-0198-9

**Published:** 2015-01-23

**Authors:** Jin Dou, Jie Tang, Chu-Hong Lu, En-She Jiang, Pei-Xi Wang

**Affiliations:** Institute of Public Health, School of Nursing, Henan University, Jinming Campus, Kaifeng, HN China 475004; Department of Preventive Medicine, School of Public Health, Guangzhou Medical University, Guangzhou, China

**Keywords:** Ischemic stroke, Suicidal ideation, Depression, Risk factor

## Abstract

**Background:**

Increasing evidences indicate that stroke confers a substantial risk for suicidal ideation. The aim of this study was to identify risk factors of suicidal ideation in acute ischemic stroke patients.

**Method:**

A total of 271 consecutive patients with acute ischemic stroke were recruited in Huai-He hospital or the First People’s Hospital, Kaifeng City, China. Demographic and clinical variables were collected and evaluated. Suicidal ideation was assessed using the Beck Scale for Suicide Ideation (BSI). Multivariate logistic regression was applied to determine the risk factors of suicidal ideation.

**Results:**

Suicidal ideation was identified in 29 patients (10.7%). It was more frequent in patients who lived in rural region, with pre-/post-stroke depression or diabetes, had a higher NIHSS score, had no confidence in disease treatment, or had a poor coping style. Living in rural region (OR 2.59, 95% CI 1.02-6.58), the presence of pre-stroke depression (OR 11.74, 95% CI 4.45-31.01), stroke severity (OR 1.20, 95% CI 1.08-1.33), having no confidence in disease treatment (OR 14.70, 95% CI 2.60-83.15), and post-stroke depression (OR 16.22, 95% CI 6.40-41.10) were independent risk factors of suicidal ideation.

**Conclusion:**

Several factors may be associated with an increased risk of suicidal ideation in acute ischemic stroke patients, including pre-/post-stroke depression, more severe stroke, having no confidence in treatment, as well as living in rural region. Our findings may have implication in risk assessment and intervention for acute ischemic stroke patients in reducing the burdens of suicidal ideation.

## Introduction

Stroke is considered one of the most devastating neurological disorders, often causing death or gross physical impairment and disability [1]. The World Health Organization (WHO) has established that 15 million people suffer from stroke worldwide each year, 5 million died and another 5 million permanently disabled [2]. Suicidal ideation (SI) is a medical term for thoughts, wishes and plans to commit suicide [3], and can vary from passive ideas to ideas with detailed plans and intent on committing suicide. Suicide has become a significant public health problem which increases morbidity and mortality and leads to serious economic burdens [4]. In China, suicide is responsible for about 287,000 deaths every year [5] and constitutes an urgent public health problem given China’s ever growing populations.

Studies on suicidal ideation in Chinese stroke patients are rare. Tang et al. [6] investigated the role of fatigue in stroke survivors and revealed that post-stroke fatigue was a significant predictor of suicidal ideation independent of depression. Chan et al. [7] found that high cerebrovascular risk factor (CVFR) scores were associated with an increased risk of suicide in middle age and older adults. Tang et al. [8] revealed that the association between insomnia (frequent awakening) and SI was significant.

Previous studies suggested that neurological disorders including stroke increase suicide risk [9-15]. The prevalence of suicidal thoughts or ideation in stroke patients ranges from 6.6% in the acute stage [16] to 10% at 3-month follow-up [9]. The assessment of the risk for suicide and the identification of risk factors for the development of suicidal thoughts and plans have important clinical implications [17]. Previous studies have shown that stroke patients often develop psychological disorders including depression, which has been consistently implicated as an important risk factor for suicide [18,19]. Studies also demonstrated that age, educational level, previous mood disorder, co-morbidities, history of stroke, disability, cognitive impairment are main risk factors for suicidal ideation in stroke patients [9-11,16]. However, additional risk factors remain to be elucidated to better guide risk assessment and monitoring of stroke patients.

The purpose of the present study was to identify new risk factors of suicidal ideation in Chinese acute ischemic stroke patients for more effective risk assessment and monitoring.

## Method

### Subjects

We recruited a total of 281 consecutive patients with first-ever or recurrent acute ischemic stroke admitted to the Neurology Department of either Huai-He Hospital or the First People’s Hospital, Kaifeng City, China from July 6, 2013 to December 6, 2013. All patients were diagnosed by a neurologist according to the fourth Chinese national conference’s recommendations on the diagnosis of cerebrovascular diseases [20]. All potential eligible patients were screened. The inclusion criteria were (1) age of 18 years or older; (2) well-documented (clinical presentation and CT brain scan) first or recurrent acute stroke occurring within the 7 days before admission [8,21]; (3) patients are consciousness (patients could identify and understand the surroundings correctly, and make accordingly, appropriate reflections, and complete the whole study questionnaire); (4) no serious complications or other systemic diseases; (5) ability and willingness to give consent. The exclusion criteria were (1) below the age of 18 years old, (2) transient ischemic attack (TIA), cerebral hemorrhage, subdural hematoma or subarachnoid hemorrhage, (3) a history of a CNS disease such as tumor, renal failure, Parkinson’s disease; (4) a history of substance abuse/dependence; (5) severe cognitive impairment defined as a Mini-Mental State Examination (MMSE) [22] score less than 20; (6) communication disorders such as aphasia [23]; (7) refused to participate in this study. Ten patients were excluded due to transient ischemic attack (TIA): 5 cases, cerebral hemorrhage: 3 cases, a history of a CNS disease such as Parkinson’s disease: 2 cases), leaving a total of 271 patients in the final study cohort.

## Assessments

### Demographic data

A demographic information collection form was prepared to include items on demographic features of the patients. Data on age, gender, marital status, educational level, residence, average family income were collected. Marital status was classified as single (including divorced) and married (including widowed). Educational level was divided into three categories according to duration of education: <= 9 years, 10–12 years, and longer than13 years. Residence was classified as urban and rural residence. Average family income was categorized into <1000 yuan/month, 1000–2000 yuan/month and >2000 yuan/month.

### Clinical information

Pre-stroke depression and cardiovascular diseases comorbidities including hypertension and diabetes were evaluated by questionnaires. Stroke severity was assessed by the National Institute of Health Stroke Scale (NIHSS) [24] by a neurologist. Stroke location was categorized as left, right, back and bilateral based on CT or MRI. The history of previous stroke including history of ischemic stroke, subarachnoidal, and intracerebral hemorrhage [9] were assessed. Physician inquired about patients’ confidence in their disease treatment by asking the question “During hospitalization, do you have any confidence in your disease treatment?” (none, partial or total). Sleep condition was classified as good, normal and poor by consulting patients or family caregivers.

## Instruments

The following assessments were performed using standard instruments at the 7th day post-stroke by a trained research assistant.

### Evaluation of suicidal ideation

Beck Scale for Suicide Ideation (BSI), which is one of the most widely used measures of suicide ideation [25], was used to determine the scale for suicide ideation (SSI). The BSI used a 19-item instrument to collect the current intensity of specific attitudes, behaviors, and plans to commit suicide. Scoring for SSI required that the participant receive a score of 1 or above for at least one of the first five items before additional items would be administered. If the participant received no score for the first five items, the total score will be zero (no ideation). As a result of this scoring method, the distribution of scores typically includes many zeroes (for non-ideators) and a more parametric distribution of scores for those who acknowledged suicidal thinking [26]. Each item has 3 choices from 0 to 2, with a total score of 0 to 38. It has a relatively high internal consistency, with a Chronbach’s alpha of 84 to 89 percent [27].

### Assessment of depression

Self-rating Depressive Scale (SDS) consisting of 20 items was evaluated according to the occurrence frequency of the symptoms. All research staff was trained. The SDS was self-administered by patients [28]. The four grades for each item were categorized as: One point (little or no time with depression symptoms); two points (part of time with the depression symptoms); three points (most time with depression symptoms); four points (almost all of the time with depression symptoms). The total scores were multiplied by 1.25 and then the integers were taken as the standard score, the lower the better. SDS index score ≥53 indicated a tendency of depression; scores 53–62, 63–72 and ≥73 represented low, mild and major depression respectively [29].

### Social support

Social Support Rating Scale (SSRS) [30] is an index of social support. The SSRS is widely cited in many countries. Additionally, it has been successfully applied to studying suicide in China [31,32]. The Scale consisted of 10 items, including objective support (three items), subjective support (four items) and the utilization of social support (three items). A higher score indicated better social support.

### Family support

Family Apgar Index was used to assess the perceived functionality of the family unit [33]. It was composed of five items: adaptation, partnership, growth, affection, and resolution, with a three-point scale ranging from 0 (hardly ever) to 2 (almost always) for each item [34]. The total scores ranged from 0 to 10, higher scores indicating higher levels of satisfaction with family functioning. A score of 0–3 indicated severe family dysfunction, 4–7 indicated moderate family dysfunction, and 8–10 indicated positive family function [35]. Cronbach’s alpha coefficients reported across studies ranged from 0.80 to 0.85 [36].

### Coping style trait

Trait Coping Style Questionnaire (TCSQ) was used to determine coping style trait into two dimensions: negative coping (NC) and positive coping (PC). The TCSQ contained 20 items, each dimension was consisted of 10 items. Coping styles were assessed by the score difference betweennegative coping and positive coping. A higher positive trait coping style score indicated that the individual’s coping style was better than average. A higher negative trait coping score suggested poor coping style [37]. If the NC score was higher than the PC score, the TCSQ was poor; if the NC score was lower than the PC score, the TCSQ was good. The validity and reliability of the TCSQ have been validated. This instrument demonstrated a good internal consistency of alpha ranged from 0.841 to 0.839 [38].

### Ethics instructions

The protocol of this study was approved by the ethics committee of Henan University. All patients were voluntarily admitted and given an informed consent.

### Statistical analysis

We categorized the presence/absence of suicidal ideation in 2 categories: without suicidal ideation and with suicidal ideation. T-test or χ2 statistics were used to assess the differences in socio-demographic and clinic factors between subjects who reported suicidal ideation and those who did not. Multivariate logistic regression models, both unadjusted and adjusted (using a forward stepwise selection strategy) were applied to analyze the risk factors of suicidal ideation. P values <0.05 were considered statistically significant. All statistical tests were performed with Statistical Package for the Social Sciences (SPSS) 13.0 (SPSS, Inc, Chicago IL).

## Results

### Demographic variables of acute ischemic stroke patients

The demographic information of acute ischemic stroke patients with and without suicidal ideation was presented in Table 1. Of the 271 patients examined, 29 (10.7%) patients reported suicidal ideation, and 242 (89.3%) did not. For the majority of demographic variables, there was no significant difference between patients with versus without SI, including age, gender, marital status, education level, average family income, and family APGAR index (all p > 0.05). However, residence in rural region was associated with an increased risk of suicidal ideation.

**Table 1:** **Demographic characteristics of suicidal ideation in acute ischemic stroke patients**

**Variables**	**With SI**	**Without SI**	p* value
	**(n = 29) (%)**	**(n = 242) (%)**	
Age, mean (SD)	65.2 (10.0)	64.4 (12.5)	0.732
Gender			
Male	17 (58.6)	129 (53.3)	0.587
Female	12 (41.4)	113 (46.7)	
Marital status			
Single	5 (17.2)	38 (15.7)	0.830
Married	24 (82.8)	204 (84.3)	
Education level			
0–9 years	24 (82.8)	201 (83.1)	0.976
10-12years	4 (13.8)	31 (12.8)	
≥13 years	1 (3.4)	10 (4.1)	
Residence			
Urban	7 (24.1)	118 (48.8)	0.012
Rural	22 (75.9)	124 (51.2)	
Average family income			
<1000 RMB/month	20 (69.0)	121 (50)	0.146
1000–2000 RMB/month	8 (27.6)	101 (41.7)	
>2000 RMB/month	1 (3.4)	20 (8.3)	
Family APGAR Index			
Severe/Moderate family dysfunction	1 (3.4)	5 (2.1)	0.633
Positive family function	28 (96.6)	237 (97.9)	

Abbreviations: *SI* Suicidal Ideation, P* values in t tests for differences in means(variable: age) or Chi-square tests for differences in proportions(other categorical variables); Severe family dysfunction, 0–3 points; Moderate family dysfunction, 4–7 points; Positive family function, 8–10 points.

### Clinical risk factors for suicidal ideation

The comparison of clinical variables in acute ischemic stroke patients with or without SI was shown in Table 2. Suicidal ideation was more frequent in patients with pre-stroke depression (χ^2^ = 39.93, *P* < 0.001), with no confidence in disease treatment (χ^2^ = 23.22, *P* < 0.001), with diabetes (χ^2^ = 4.56, *P* = 0.033), with poor TCSQ (χ^2^ = 13.62, *P* < 0.001) or with post-stroke depression (χ^2^ = 66.21, *P* <0 .001). Furthermore, NIHSS score was associated with suicidal ideation (P < 0.001).

**Table 2:** **Clinical factors related to suicidal ideation in acute ischemic stroke patients**

**Variables**	**With SI**	**Without SI**	p* value
	**(n = 29) (%)**	**(n = 242) (%)**	
Pre-stroke depression			
Yes	15 (51.7)	22 (9.1)	<0.001
No	14 (48.3)	220 (90.9)	
Comorbidities			
Hypertension	16 (55.2)	138 (57.0)	0.849
Diabetes	9 (31.0)	37 (15.3)	0.033
Heart disease	4 (13.8)	31 (12.8)	0.881
NIHSS score, mean (SD)	5.3 (4.9)	2.1 (2.7)	<0.001
Post-stroke depression			
Normal	7 (24.1)	203 (83.9)	<0.001
Mild depression	13 (44.8)	33 (13.6)	
Moderate/Major depression	9 (31.0)	6 (2.5)	
Stroke location			
Left(front)	5 (17.2)	35 (14.5)	0.967
Right(front)	5 (17.2)	47 (19.4)	
Bilateral(front)	6 (20.7)	55 (22.7)	
Back ( left , right and bilateral)	13 (44.8)	105 (43.4)	
Disease treatment confidence			
None	4 (13.8)	4 (1.7)	<0.001
Partial	9 (31.0)	28 (11.6)	
Totally	16 (55.2)	210 (86.8)	
History of stroke			
Yes	12 (41.4)	96 (39.7)	0.859
No	17 (58.6)	146 (60.3)	
Sleep condition			
Good	8 (27.6)	99 (40.9)	0.186
Normal	8 (27.6)	73 (30.2)	
Poor	13 (44.8)	70 (28.9)	
SSRS score, mean (SD)	36.9 (4.6)	38.0 (4.8)	0.253
TCSQ			
Poor	16 (55.2)	56 (23.1)	<0.001
Good	13 (44.8)	186 (76.9)	

*Note:* NIHSS, National Institute of Health Stroke Scale; SDS, Self-rating Depression Scale; SSRS, Social Support Rate Scale; TCSQ, Trait Coping Style Questionnaire; P* values in t tests for differences in means (variable: NIHSS score) or Chi-square tests for differences in proportions (other categorical variables).

### Logistic regression analyses

We performed a stepwise logistic regression analysis with residence, pre-stroke depression, confidence in disease treatment, diabetes, NIHSS score, post-stroke depression, TCQS identified as significant predictor variables (Table 3). Residence in rural region (OR = 2.59, 95% CI 1.02-6.58), pre-stroke depression (OR = 11.74, 95% CI 4.45-31.01), having no confidence in disease treatment (OR = 14.70, 95% CI 2.60-83.15), stroke severity (OR = 1.20, 95% CI 1.08-1.33) and post-stroke depression (OR = 16.22, 95% CI 6.40-41.10) were significant risk factors for suicidal ideation in acute ischemic stroke patients.

**Table 3:** **Multivariate logistic regression analysis of significant risk factors for suicidal ideation in acute ischemic stroke patients**

Variables*	***OR***	**95% CI**	***p value***
Residence			
Urban	reference		
Rural	2.59	1.02-6.58	0.045
Pre-stroke depression			
Yes	11.74	4.45-31.01	<0.001
No	reference		
Disease treatment confidence			
None	14.70	2.60-83.15	0.002
Partial	4.17	1.41-12.33	0.010
Totally	reference		
NIHSS score	1.20	1.08-1.33	0.001
Post-stroke depression			
No	reference		
Yes	16.22	6.40-41.10	<0.001

*Variables considered for inclusion in the regression model were age, gender, marital status, education level, residence, average family income, pre-stroke depression, comorbidities, NIHSS score, post-stroke depression, disease treatment confidence and TCSQ. Only significant predictor variable were included in the final model.

OR, *Odds Ratio;* CI, confidence interval.

## Discussion

Suicide in stroke patients remains a huge public health burden. Better understanding about factors influencing suicidal ideation in stroke patients is critical in risk assessment, monitoring and interventions to reduce its burdens.

To the best of our knowledge, studies on suicidal ideation in stroke patients in china are rare. Our study identified several risk factors of suicidal ideation, including pre- or post-stroke depression, more severe stroke, having no confidence in disease treatment, as well as living in rural region. Our findings provide new etiology insights as well as practical implications for stroke patients.

In our study, 10.7% stroke patients reported suicidal ideation, while the prevalence of suicidal ideation in stroke patients ranged from 6.6% in the acute stage to 10% at the 3-month follow-up in previous studies [9,16]. It has been well established that people who survive stroke often develop post-stroke depression, which has been consistently implicated as an important risk factor for suicide [18,19,39]. Our study validated this relationship. Moreover, we identified that pre-stroke depression was also an important factor influencing suicidal ideation in stroke patients. This is consistent with a recent population-based study demonstrating that pre-stroke depression increased the risk of suicide [40].

The place of residence is an interesting factor in developed countries; the suicide rate of rural residents is far lower than urban residents. However, in China, rural residents have 3 to 5 times higher suicide rate than urban residents [8]. In Chinese rural regions, due to poorer socioeconomic development, stroke patients endue heavier financial burdens and greater psychological pressure; this may explain their higher risk of suicidal ideation. In this study, we confirmed that living in rural region significantly increase the risk of suicidal ideation in stroke patients.

Rao et al. [41] found that individuals with cerebrovascular disease were at an increased risk of suicide. Chan et al. [7] found that high cerebrovascular risk factor (CVFR) scores were associated with an increased risk of suicide in stroke patients. Our research confirmed that stroke comorbidity diabetes mellitus could significantly increase the risk of suicide ideation in acute ischemic stroke patients, as in the studies of Santos et al. [10] and Pompili et al. [11].

We also tested whether patients’ physical function is an important factor in influencing suicide ideation. In this study, physical function was evaluated by the National Institute of Health Stroke Scale (NIHSS). Our analysis revealed that physical function was an important factor for suicidal ideation in stroke patients. Additionally, through the question “During hospitalization, do you have any confidence in your disease treatment?”, we revealed that stroke patients with no confidence in treatment had an increased risk of suicidal ideation. Moreover, patients’ coping style significantly influenced the risk of suicidal ideation.

### Innovation and weakness of the study

Our study is innovative in that we assessed various demographic and clinical factors in our analyses. To the best of our knowledge, we discovered for the first time that both pre and post-stroke depression, more severe stroke, having no confidence in disease treatment, as well as living in rural region could significant influence the risk of suicidal ideation in acute ischemic stroke patients in China .There are also study limitations. The cross-sectional study design limits the evidence of causality. The number of SI patients was relatively small (n = 29). Further prospective studies with larger sample sizes are warranted to confirm our findings.

## Conclusions

We identified that several risk factors of suicidal ideation in Chinese acute ischemic stroke patients. These findings may have implication in risk assessment and monitoring of acute ischemic stroke patients to reduce the burdens of suicidal ideation in China.
